# *Helicobacter pylori* cholesterol-α-glucosyltransferase manipulates cholesterol for bacterial adherence to gastric epithelial cells

**DOI:** 10.1080/21505594.2021.1969171

**Published:** 2021-09-10

**Authors:** Chung-Yao Hsu, Jia-Yin Yeh, Chun-Ya Chen, Hui-Yu Wu, Meng-Hsuan Chiang, Chia-Lin Wu, Hwai-Jeng Lin, Cheng-Hsun Chiu, Chih-Ho Lai

**Affiliations:** a Graduate Institute of Biomedical Sciences, Department of Microbiology and Immunology, Department of Biochemistry, College of Medicine, Chang Gung University, Taoyuan, Taiwan; b Department of Laboratory Medicine, Taichung Veterans General Hospital Chiayi Branch, Chiayi, Taiwan; c Molecular Infectious Disease Research Center, Department of Pediatrics, Department of Neurology, Chang Gung Memorial Hospital, Linkou, Taiwan; d Division of Gastroenterology and Hepatology, Department of Internal Medicine, Shuang-Ho Hospital, New Taipei, Taiwan; e Division of Gastroenterology and Hepatology, Department of Internal Medicine, School of Medicine, College of Medicine, Taipei Medical University, Taipei, Taiwan; f Department of Microbiology, School of Medicine, China Medical University, Taichung, Taiwan; g Department of Nursing, Asia University, Taichung, Taiwan

**Keywords:** *Helicobacter pylori*, cholesterol glucosylation, phosphatidylserine, inflammation

## Abstract

*Helicobacter pylori* infection is associated with several gastrointestinal diseases, including gastritis, peptic ulcers, and gastric cancer. Infection of cells with *H. pylori* is dependent on lipid rafts, which are cholesterol-rich microdomains located in the cell membrane. *H. pylori* cholesterol-α-glucosyltransferase (CGT) catalyzes the conversion of membrane cholesterol to cholesteryl glucosides, which can be incorporated into the bacterial cell wall, facilitating evasion from immune defense and colonization in the host. However, the detailed mechanisms underlying this process remain to be explored. In this study, we discovered for the first time that *H. pylori* CGT could promote adherence to gastric epithelial cells in a cholesterol-dependent manner. Externalization of cell membrane phosphatidylserine (PS) is crucial for enhancement of binding of *H. pylori* to cells by CGT and for cytotoxin-associated gene A (CagA)-induced pathogenesis. Furthermore, exogenous cholesterol interferes with the actions of *H. pylori* CGT to catalyze cellular cholesterol, which impedes bacterial binding to cells and attenuates subsequent inflammation, indicating that the initial attachment of *H. pylori* to cells is closely dependent on host cholesterol. These results provide evidence that CGT contributes to *H. pylori* infectivity and it may serve as a key target for the treatment of *H. pylori*-associated diseases.

## Introduction

*Helicobacter pylori*, a gram-negative microaerophilic bacterium, usually colonizes the human stomach. *H. pylori* infection is associated with a high incidence of gastrointestinal diseases, including gastritis, peptic ulcers, and gastric cancer [1]. Several important virulence factors have been found to contribute to *H. pylori* pathogenesis, including vacuolating cytotoxin A (VacA) and cytotoxin-associated gene A (CagA) [2]. VacA is classified as a pore-forming toxin that possesses the capacity to stimulate intracellular acidic vacuole formation and disrupt cellular homeostasis, leading to apoptosis [3]. CagA can be translocated by the *H. pylori* type IV secretion system (TFSS) upon attachment to cells, causing chronic inflammation and oncogenesis in gastric epithelial cells [4].

Other adhesion molecules have also been discovered in *H. pylori*, such as Le^b^ blood group antigen binding adhesin (BabA) [5] and sialic acid–binding adhesin (SabA) [6], which bind to Lewis antigens and gangliosides that are expressed in human gastric epithelial cells [7,8]. In addition to adhesion molecules, cholesterol-α-glucosyltransferase (CGT) has been found to consolidate bacterial colonization in mouse models [9]. CGT is mainly located on the bacterial inner membrane and is encoded by *hp0421* [10]. *H. pylori* takes up host cholesterol, which is then converted into cholesterol-α-D-glucopyranoside (αCG), which is further modified to cholesteryl-6-O-tetradecanoyl-α-D-glucopyranoside (αCAG) and cholesteryl-6-O-phosphatidyl-α- D-glucopyranoside (αCPG) [9–13]. The cholesteryl glucosides can be recruited to the *H. pylori* cell wall, which is closely associated with immune evasion [9,14].

Lipid rafts are membrane cholesterol-rich microdomains that provide entry portals for many bacterial pathogens or their virulence factors [15]. Both CagA translocation and VacA delivery into host cells following *H. pylori* infection require lipid rafts [16–18]. *H. pylori* exploits host externalized phosphatidylserine (PS) for CagA delivery *via* TFSS and it subsequently induces pathogenesis [19]. These results are in accordance with the finding that *cgt*-deficient *H. pylori* show a reduced ability to introduce CagA by TFSS [20]. However, detailed information on the relationship between CGT and membrane PS in response to *H. pylori* colonization of host cells is required for further studies. This study investigated how *H. pylori* CGT manipulates cellular cholesterol and membrane PS, which contribute to the bacterial pathogenesis of the host. Furthermore, *H. pylori* CGT-enhanced bacterial adherence could be dampened by the addition of soluble cholesterol, which elucidated the different strategies for controlling *H. pylori* infection in the clinic.

## Results

### H. pylori CGT promotes bacterial adherence to cells

**Figure 1.**
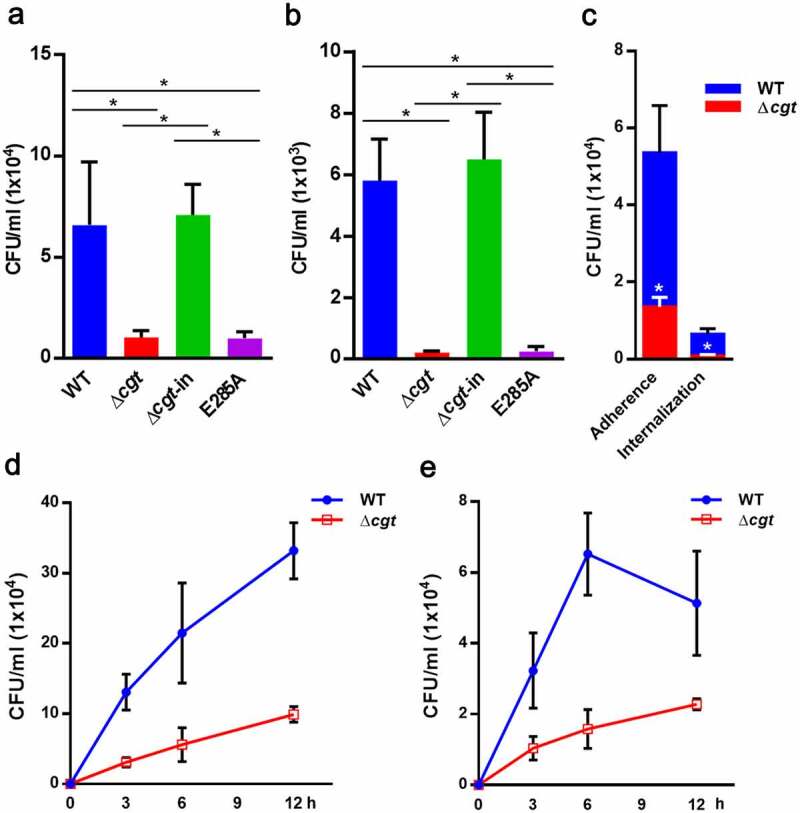
*H. pylori* CGT is essential for bacterial adherence to cells. AGS cells were infected with WT, ∆*cgt*, ∆*cgt*-in, and *cgt* dead-mutant *H. pylori* at a MOI of 50 for 6 h. *H. pylori* (a) adhesion to and (b) invasion of cells were analyzed and expressed as CFU. (c) Competition assay involving co-infection with WT and ∆*cgt H. pylori* (1:1) was conducted. Cells where viable WT and ∆*cgt H. pylori* both adhered to and invaded, or only invaded were enumerated, and plated onto chloramphenicol (34 μg/ml) supplemented blood agar plates to select ∆*cgt H. pylori*. Cells were infected with *H. pylori* at a MOI of 100 for 3, 6, and 12 h, and (d) adhesion and (e) invasion assays were performed. *, *P*< 0.05

**Figure 2.**
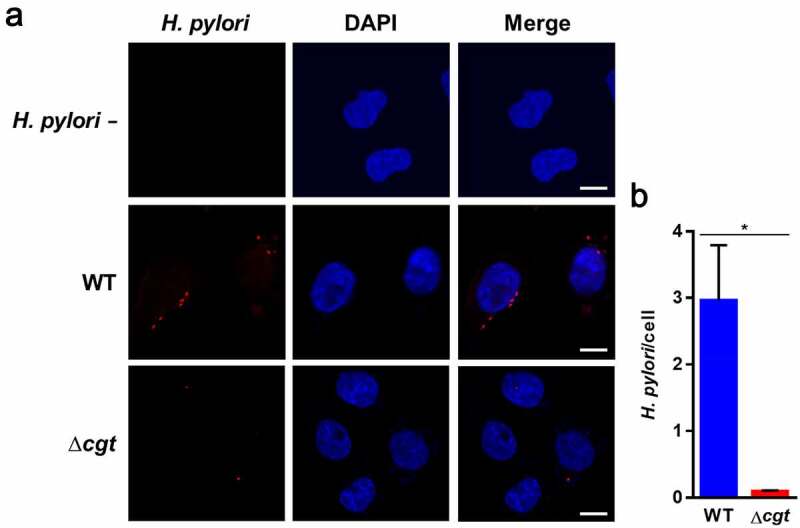
Mutation of CGT reduces *H. pylori* binding to AGS cells. (a) AGS cells were uninfected or infected with WT or ∆*cgt H. pylori* at a MOI of 20 for 6 h. Cells were probed with anti-*H. pylori* antibody (red) and stained with DAPI (blue) to visualize cell nuclei. The stained samples were analyzed using confocal microscopy. Scale bar, 5 μm. (b) Total *H. pylori* were enumerated and divided by the number of AGS cells in each field. The mean number of bacteria was calculated using three different fields. *, *P*< 0.05

*H. pylori* CGT is involved in pathogenesis through mechanisms such as host colonization [13] and CagA translocation [20]. *H. pylori* adherence to cells is the initial step in the infection of host cells and it is essential for CagA translocation *via* TFSS. To investigate whether *H. pylori* CGT affects *H. pylori* attachment to gastric epithelial cells, we constructed *H. pylori* isogenic mutants, including *cgt* knockout (∆*cgt), cgt* knockin (∆*cgt*-in), and *cgt* dead-mutant (E285A) (Fig. S1). AGS cells (human gastric adenocarcinoma cell line) were infected with *H. pylori* WT or isogenic mutants for 6 h, followed by adhesion and invasion assays. Figure 1a and 1b show that the adhesion and invasion activities of WT and ∆*cgt*-in cells were much higher than those of ∆*cgt* and *cgt*-dead mutant strains. These results were also supported by the results of competition experiment involving co-infection with WT and ∆*cgt H. pylori* (1:1) (Figure 1c), indicating that CGT-bearing *H. pylori* is superior in bacterial attachment and internalization to gastric epithelial cells. We further conducted a time-course analysis of the bacterial adhesion assay (Figure 1d) and internalization assay (Figure 1e). Our results showed that WT *H. pylori* possesses higher adherence and intracellular survival than ∆*cgt* in a time-dependent manner. We then investigated *H. pylori* localization in infected cells using immunofluorescence microscopy. As shown in Figure 2, WT *H. pylori* adhering to AGS cells was much greater than that of ∆*cgt*. These results demonstrate that CGT promotes *H. pylori* adhesion to gastric epithelial cells, thereby contributing to its internalization by cells.

### Disruption of lipid rafts decreases H. pylori infection of cells

**Figure 3.**
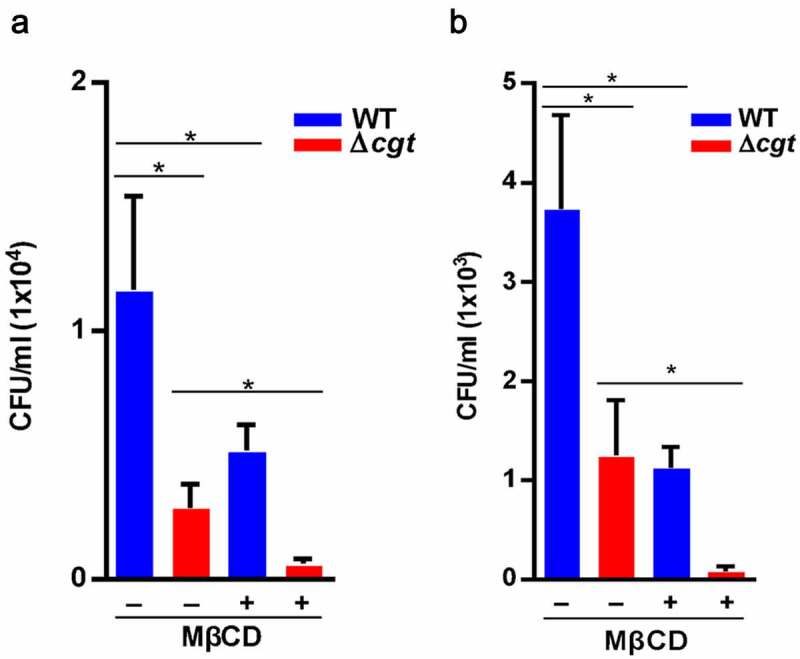
Involvement of lipid rafts in *H. pylori* infection. (a) AGS cells were untreated or pretreated with 5 mM MβCD for 30 min, followed by infection with WT or ∆*cgt H. pylori* at a MOI of 100 for 6 h. *H. pylori* (A) adhesion to and (b) invasion of cells were analyzed. *, *P*< 0.05

**Figure 4.**
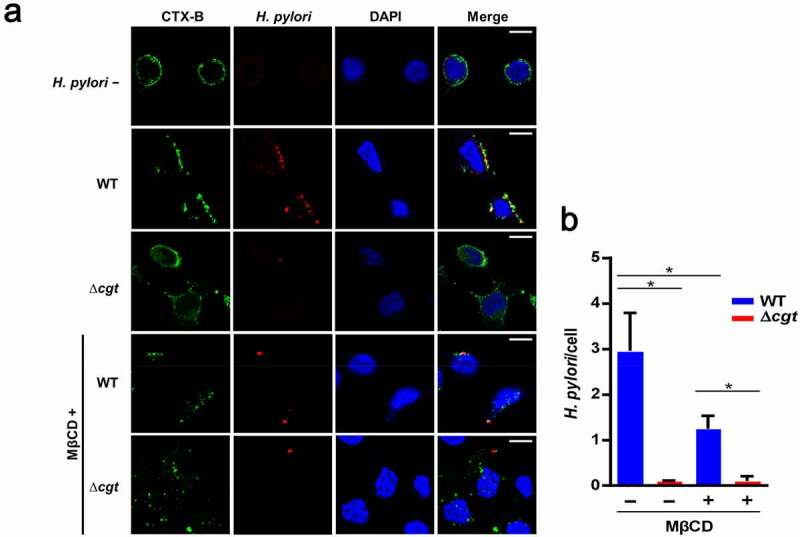
*H. pylori* CGT coalesces lipid rafts on the cell membrane. (a) AGS cells were untreated or pretreated with 5 mM MβCD for 30 minutes, followed by *H. pylori* infection at a MOI of 20 for 6 h. Cells were probed with anti-*H. pylori* antibody (red) and FITC-CTX-B to visualize GM1 (green), and DAPI (blue) to visualize cell nuclei. The stained samples were analyzed by confocal microscopy. Scale bar, 5 μm. (b) Total number of *H. pylori* were counted and divided by the number of AGS cells untreated or pretreated with MβCD in each field. The mean number was calculated using three different fields. *, *P*< 0.05

Since lipid rafts play a pivotal role in *H. pylori* pathogenesis in gastric epithelial cells [17], we next explored whether CGT is involved in this process. AGS cells were pretreated with a cholesterol-depleting agent, methyl-β-cyclodextrin (MβCD), prior to infection with WT or ∆*cgt H. pylori*. MβCD pretreatment decreased bacterial adhesion to cells in both the WT and ∆*cgt* strains (Figure 3a). Similarly, the intracellular survival rate was also reduced in MβCD-pretreated cells (Figure 3b). We then employed immunofluorescence microscopy to investigate whether disruption of lipid rafts by MβCD inhibits *H. pylori* infection in cells. As shown in Figure 4, raft clustering resulted from WT *H. pylori* infection, but not from ∆*cgt*. Notably, the number of WT *H. pylori* attached to the cell membrane was markedly decreased in MβCD-pretreated cells. These results demonstrate that both CGT and the integrity of lipid rafts are essential for *H. pylori* infection.

### PS externalization is the consequence of H. pylori adhesion

**Figure 5.**
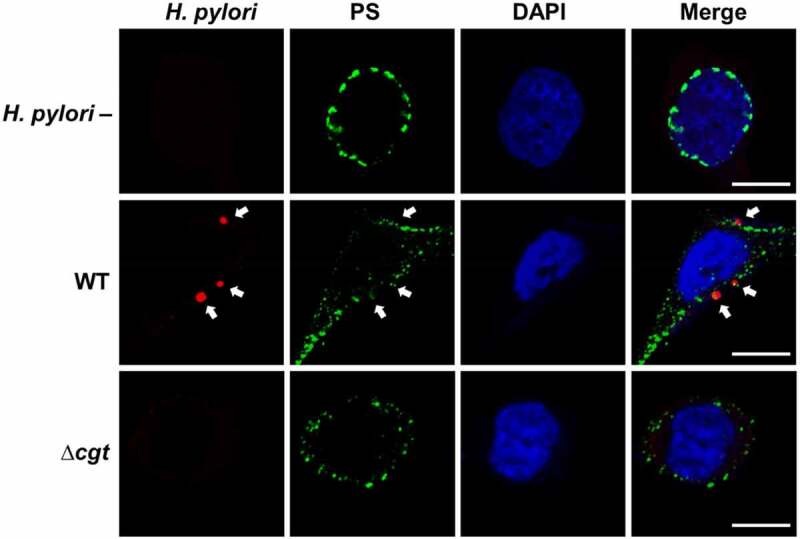
*H. pylori* co-localizes with externalized PS. AGS cells were uninfected or infected with *H. pylori* at a MOI of 20 for 6 h. Cells were probed with anti-*H. pylori* antibody (red) or stained with anti-PS antibody (green), and DAPI to visualize cell nuclei (blue). The stained cells were analyzed by confocal microscopy. Arrows indicated *H. pylori* attachment to cell membrane and colocalization with PS. Scale bar, 5 μm

**Figure 6.**
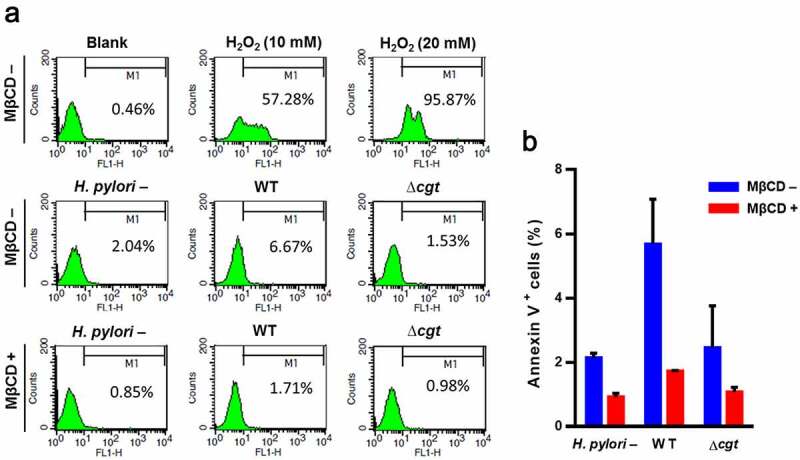
*H. pylori* CGT triggers membrane PS externalization. (a) AGS cells were untreated or pretreated with 5 mM MβCD for 30 min, followed by infection with WT or ∆*cgt H. pylori* at a MOI of 100 for 6 h. The cells were probed with anti-PS antibody, and analyzed *via* flow cytometry. Cells were treated with H_2_O_2_ (10 or 20 mM) for 4 h as a positive control. (b) The level of membrane PS externalization was determined

*H. pylori* infection triggers non-apoptotic externalization of PS, which interacts with CagA, resulting in CagA translocation [19]. In addition, CGT-bearing *H. pylori* has been shown to enhance CagA translocation [20]. Using confocal microscopy, we further analyzed whether *H. pylori* CGT regulates translocation of CagA by altering cellular PS. Figure 5 shows that ∆*cgt* adheres to cells much less than the WT strain. Externalization of membrane PS during *H. pylori* infection was assessed using flow cytometry. As shown in Figure 6, WT *H. pylori* triggered more PS externalization (6.7%) than that resulting from ∆*cgt* infection (1.5%). When cells were pretreated with MβCD followed by infection with WT *H. pylori*, a decrease in PS externalization occurred compared to that in untreated cells. Although PS externalization was reduced in MβCD-treated AGS cells infected with ∆*cgt*, there was no significant difference between the MβCD treatment and the untreated control. These results indicate that PS externalization is essential for the CGT-promoted adhesion of *H. pylori* to cells.

### Cholesterol coating of H. pylori inhibits its adhesion to cells

**Figure 7.**
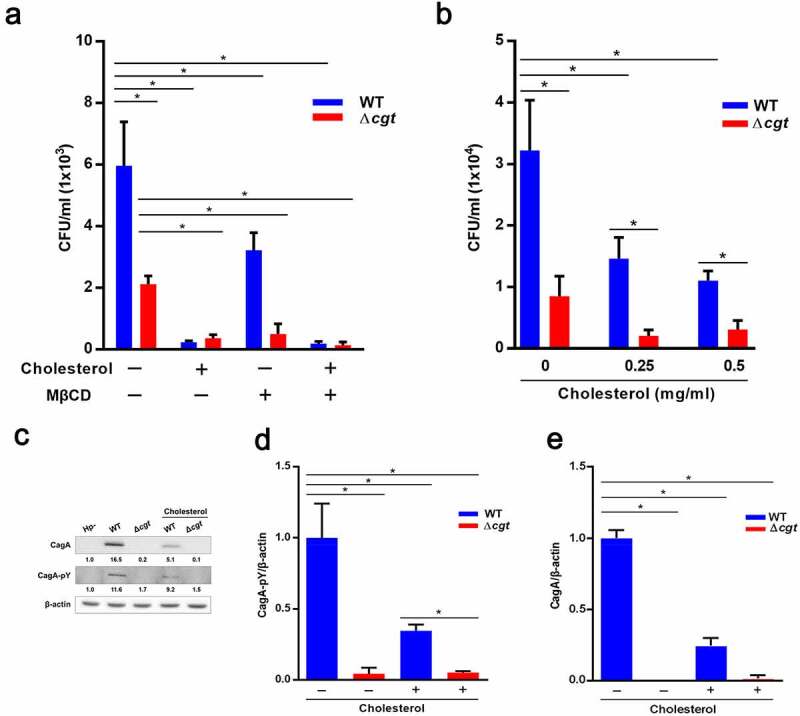
Exogenous cholesterol coating of *H. pylori* decreases its adhesion to cells. (a) *H. pylori* were untreated or pretreated with water-soluble cholesterol (5 mg/ml) for 1 h before infection. AGS cells pretreated with 5 mM MβCD for 30 min were then infected with WT or Δ*cgt H. pylori* at a MOI of 100 for 6 h. Viable *H. pylori* that had either adhered to or invaded AGS cells were analyzed. (b) AGS cells were infected with WT or ∆*cgt H. pylori* at MOI of 100 for 6 h that were untreated or pretreated with water-soluble cholesterol (0.25 and 0.5 mg/ml) for 1 h prior to infection. Viable *H. pylori* that had either adhered to or invaded AGS cells were analyzed. (c) AGS cells were infected with WT or ∆*cgt H. pylori* at MOI of 100 for 6 h. *H. pylori* were pretreated with water-soluble cholesterol (5 mg/ml) for 1 h before infection. The levels of CagA translocation and phosphorylation were assessed using a western blot assay. β-actin was an internal control and relative protein levels were normalized to those in the uninfected group. (d) CagA phosphorylation and (e) translocation were compared between each condition and expressed as fold changes. *, *P*< 0.05

As CGT-containing *H. pylori* coated with excessive cholesterol influences inflammatory signaling [13], we further tested whether exogenous cholesterol affects CGT-mediated *H. pylori* adhesion to cells. Our results showed that coating *H. pylori* with water-soluble cholesterol (5 mg/mL) did not affect the bacterial viability (Fig. S2). Treatment of bacteria with exogenous cholesterol markedly decreased WT adherence to AGS cells compared to that of non-cholesterol-coated *H. pylori* (Figure 7a). Similar results were obtained for the ∆*cgt* strain but with less reduction. When higher concentrations of cholesterol were added (0.25 and 0.5 mg/mL), a significant decrease in WT *H. pylori* adhesion to cells was observed compared with that in cholesterol-untreated cells (Figure 7b). Additionally, treatment with water-soluble cholesterol led to remarkable inhibition of *H. pylori* CagA translocation/phosphorylation (Figure 7c-e), NF-κB activation (Fig. S3A), and IL-8 production (Fig. S3B). This trend was also observed in the ∆*cgt* strain, but with only a slight effect. The results indicate that the initial attachment of *H. pylori* to cells is dependent on host cholesterol, while exogenous cholesterol competes with *H. pylori* CGT actions to catalyze cellular cholesterol, thereby dampening its binding to cells. Taken together, our findings demonstrate that CGT contributes to the initial binding of *H. pylori* to cells, which is crucial for promoting subsequent pathogenesis.

## Discussion

*H. pylori* infection exploits PS for CagA translocation [19] and CGT is involved in this process in a raft-dependent manner [20]. Although PS externalization plays a pivotal role in *H. pylori* CagA translocation, the effect of CGT on bacterial adherence and inflammatory responses remains to be explored. In this study, we found that *H. pylori* CGT elicits membrane cholesterol coalescence, which promotes bacterial adherence to cells, leading to subsequent pathogenesis. Unveiling the mechanism of bacterial virulence factors is particularly crucial, as targeting of key molecules to treat *H. pylori*-associated diseases has been proposed.

*H. pylori* CGT converts cholesterol into cholesterol-α-D-glucopyranoside (αCG), cholesteryl-6ʹ-α-*O*-tetradecanoyl-α-D-glucopyranoside (αCAG), and cholesteryl-6ʹ-α-*O*- phosphatidyl-α-D-glucopyranoside (αCPG), which are categorized as cholesteryl glucosides [10]. Glucoside derivatives have been shown to generate anti-inflammatory responses [21–24]. Notably, *H. pylori* utilizes cholesteryl glucosides catalyzed by CGT to evade the host immune system, which is beneficial for its survival in host cells [9,25]. Furthermore, cholesterol glucosylation by *H. pylori* is crucial for the manipulation of autophagy to delay the macrophage clearance of bacteria [26]. A recent study reported that cholesteryl α-D-glucopyranoside acyltransferase and CGT are packaged in outer membrane vesicles and delivered to host cells, followed by gathering raft-associated molecules, to enhance bacterial attachment to cells [27]. The present study further showed that CGT in *H. pylori* not only enhanced its adherence ability, but also increased its intracellular survival in gastric epithelial cells. In addition, *H. pylori* itself expresses flotillin-like protein and Lewis antigens that serve as an immune mimicry to the host, providing an alternative way to evade host immune surveillance [14,28,29]. Together with previous findings, our current study reveals that the mechanisms underlying *H. pylori* infection involve action of CGT to orchestrate immune defense *via* cholesterol glucosylation, resulting in successful host survival.

*H. pylori* is much more likely to induce host pathogenesis by utilizing CGT to sense cholesterol-rich regions such as membrane lipid rafts, which mediate many responses during *H. pylori* infection [9]. However, a recent study indicated that *H. pylori* coated with excess cholesterol prior to infection failed to block the IFNγ-mediated inflammatory pathway [13]. In our study, treatment of CGT-bearing *H. pylori* with water-soluble cholesterol showed a decrease in its adherence to AGS cells when compared to that of *H. pylori* not coated with cholesterol. The binding of *H. pylori* to cells is largely dependent on host cholesterol, while exogenous cholesterol-coated *H. pylori* competes with CGT to modify host cholesterol to generate cholesteryl glucosides, resulting in decreased bacterial adhesion to cells. These results indicate that the source of cholesterol is important for *H. pylori* CGT functions, which contributes to the initial binding of bacteria to cells and subsequent pathogenesis.

Several molecules have been found in membrane lipid rafts that are involved in the adhesion of *H. pylori* to cells, including PAR1/MARK, Lewis antigens, and integrin α5β1 [30,31]. Depletion of cholesterol affects membrane composition, which has an indirect effect on the ability of *H. pylori* to adhere to cells. This phenomenon motivated us to further investigate whether a mutation of CGT in *H. pylori* could exert a direct inhibitory effect on bacterial adhesion to cells. CGT catalyzes the conversion of cholesterol to cholesteryl glucosides, which are then incorporated into the bacterial cell wall, and ∆*cgt* cannot catalyze this conversion [9]. Our results showed that without MβCD treatment, ∆*cgt* significantly decreased the binding of *H. pylori* to cells compared with that of the wild-type strain. These results excluded the effects of MβCD on membrane proteins and demonstrated that the abrogation of cholesterol glucosylation reduces the ability of *H. pylori* to adhere to cells. Our findings are supported by previous studies showing that the dependence on cholesterol is quite important [32,33]. This is because ∆*cgt* is unable to glucosylate cholesterol, leading to decreased colonization in murine models [13].

Bacterial virulence factors employing membrane cholesterol microdomains to gain access to host cells have been reported previously [34–36]. Cholesterol usurping/depleting agents have been used to alleviate infectious diseases by preventing microbial entry into host cells [37]. For instance, cholesterol-lowering agents (i.e., statins) potentially attenuate pathogen infectivity [38–41]. Another cholesterol-depleting agent, MβCD, is commonly employed to reduce microbial adherence to the host cell membrane [36,42,43]. Our previous studies demonstrated that statin use significantly reduced the incidence of *H. pylori*-related diseases [41,44,45]. Moreover, hijacking cholesterol by antagonists competed for VacA and CagA actions *via* lipid rafts alleviated *H. pylori*-induced pathogenesis [46]. Consistent with these findings, the present study showed that depletion of cholesterol interferes with *H. pylori* adherence and internalization into cells, which depends on CGT. These lines of evidence suggest that pharmaceutical targeting of lipid rafts should be developed to treat *H. pylori* infection.

Although the *in vitro* cell models have demonstrated that CGT contributes to *H. pylori* adherence to gastric epithelial cells, some limitations exist in the present study. For example, only the AGS cell line was used as a cell-based assay platform. Although AGS cells have been well studied in the *H. pylori* infection models, more gastric cell lines should be considered to validate the findings. In addition, the direct linkage between cholesterol, CGT, and *H. pylori* infectivity using long-term animal or *in vivo* studies deserves further investigations.

**Figure 8.**
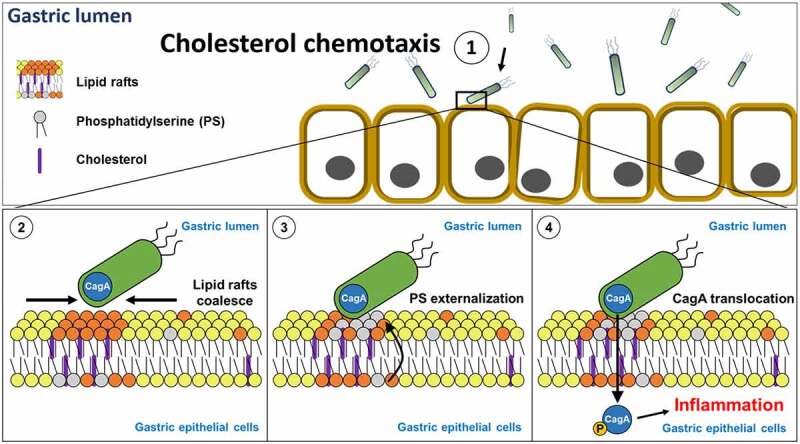
Hypothesized model depicts that CGT is crucial for *H. pylori* adhesion to gastric epithelial cells and subsequent pathogenesis. (1) WT (*cgt^+^*) *H. pylori* utilizes cholesterol to adhere to cholesterol-rich microdomains in cell membrane. (2) *H. pylori* coalesces lipid rafts, then (3) triggers non-apoptotic PS externalization to (4) enhance CagA translocation *via* TFSS, leading to inflammation and pathogenesis of host cells

In summary, this study demonstrates that *H. pylori* CGT drives cholesterol glucosylation, which is pivotal for the exploitation of lipid rafts as a foothold in its initial attachment to cells. Moreover, membrane PS externalization is essential for CGT-promoting *H. pylori* adhesion to cells, followed by CagA translocation and phosphorylation (Figure 8). The results further show that exogenous cholesterol competes with CGT actions in modifying cellular cholesterol, which decreases bacterial adherence and inflammation of cells. Exploring the mechanism of how CGT contributes to *H. pylori* infectivity might provide an opportunity to develop new agents to alleviate its pathogenesis.

## Materials and methods

### Cell culture

AGS cells (ATCC CRL-1739) were cultured in F12 medium (Thermo Fisher Scientific, Waltham, MA, USA) containing 10% fetal bovine serum (Hyclone, Logan, UT, USA). Antibiotics were not added to the cell culture medium in the *H. pylori*-infected assay. Cells were cultured at 37°C in a 5% CO_2_ atmosphere.

### Bacterial culture

*H. pylori* 26,695 (ATCC 700392) wild-type (WT), *cgt* knockout (∆*cgt), cgt* knockin (∆*cgt*-in), and *cgt* dead-mutant (E285A) strains were constructed (Fig. S1). Each isogenic mutant *H. pylori* was generated by the insertion of an antibiotic resistance cassette *via* allelic replacement, as described previously [26]. *H. pylori* wild-type and isogenic mutants were routinely cultured on blood agar plates (Brucella agar with 10% defibrinated sheep blood) and incubated in a microaerophilic environment (10% CO_2_, 5% O_2_, 85% N_2_) at 37°C.

### Gentamycin protection assay

AGS cells (2 × 10^5^) were seeded in 12-well plates and cultured for 16 h. The cells were treated with 5 mM methyl-β-cyclodextrin (MβCD) (Sigma-Aldrich, St. Louis, MO, USA) for 30 min, followed by infection with *H. pylori* strains (WT, ∆*cgt*, and E285A) at an MOI of 100 for 3, 6, and 12 h. The infected cells were washed with PBS prior to incubation with 100 μg/mL gentamycin (Sigma-Aldrich) for 90 min. The cells were then lysed using sterile water. The cell lysate was serially diluted onto blood agar plates and incubated for 5 days. Viable *H. pylori* were counted and indicated as colony-forming units (CFUs).

### Immunofluorescence staining and confocal microscopic analysis

AGS cells (2 × 10^5^) were seeded in 6-well plates and cultured for 16 h. The cells were treated with 5 mM MβCD for 30 min prior to *H. pylori* infection at an MOI of 20 for 6 h. Then, the cells were fixed with 1% paraformaldehyde (Alfa Aesar, Haverhill, MA, USA) for 1 h, permeabilized with 0.1% Triton X-100 for 30 min, and blocked with 1% FBS for 1 h. The cells were probed with an anti-*H. pylori* antibody (Bio-Techne, Minneapolis, MN, USA) and anti-phosphatidylserine antibodies (MERCK, Darmstadt, Germany), respectively. The nuclei and membrane lipid rafts were stained with 4ʹ,6-diamidino-2-phenylindole (DAPI) and fluorescein isothiocyanate-conjugated cholera toxin subunit B (FITC-CTX-B) (Invitrogen, Carlsbad, CA, USA). The fluorescent signals were analyzed using a confocal laser scanning microscope (LSM780, Carl Zeiss, Oberkochen, Germany).

### Flow cytometry

AGS cells (2 × 10^5^) were seeded in 6-cm plates and cultured for 16 h. The cells were treated with 5 mM MβCD for 30 min, followed by WT or ∆*cgt H. pylori* infection at an MOI of 100 for 6 h. The infected cells were washed with PBS and probed with an anti-phosphatidylserine antibody at room temperature for 1 h. The cells were incubated with FITC-conjugated goat anti-mouse IgG1 antibody at room temperature for 30 min. The stained cells were determined using a FACSCalibur™ flow cytometer (Becton, Dickinson and Company, Franklin Lakes, NJ, USA) and analyzed using the Cell Quest software WinMDI (Verity Software House, Topsham, ME, USA).

### Western blot assay

*H. pylori* were pretreated with water-soluble cholesterol (5 mg/mL) for 1 h prior to infection. AGS cells were treated with 5 mM MβCD for 30 min, followed by infection with WT or Δ*cgt H. pylori* at an MOI of 100 for 6 h. *H. pylori*-infected cells were analyzed by 6% SDS-PAGE followed by transfer onto polyvinylidene difluoride membranes (Millipore, Burlington, MA, USA). The membranes were blocked with 5% skim milk for 1 h prior to incubation with the primary antibody at 4°C overnight. The membrane was then incubated with a horseradish peroxidase-conjugated secondary antibody (Millipore). Protein expression levels were analyzed using ECL Western Blotting Detection Reagents (GE Healthcare, Chicago, IL, USA) and analyzed using the Azure 400 system and AzureSpot Analysis Software (Azure Biosystems, Dublin, CA, USA).

### Statistical analysis

Experimental results are expressed as the mean ± standard deviation of independent triplicate experiments. Student’s *t*-test was used to calculate the statistical significance of differences between the two groups. Differences were considered significant at *P*< 0.05. Statistical analysis was performed using Prism6 (Graph Pad, San Diego, CA, USA).

## Supplementary Material

Supplemental Material

## Data Availability

The authors confirm that the data supporting the findings of this study are available within the article and its supplementary materials. https://doi.org/10.1080/21505594.2021.1969171
