# Supplementary material for: Helicobacter pylori cholesterol-α-glucosyltransferase manipulates cholesterol for bacterial adherence to gastric epithelial cells
Source: Virulence. 2021 Sep 10;12(1):2341–51. doi: 10.1080/21505594.2021.1969171 (PMC8437457; doi:10.1080/21505594.2021.1969171)
Supplement: Supplemental Material [file KVIR_A_1969171_SM1032.zip › spp.pdf]

# Supplementary Figures

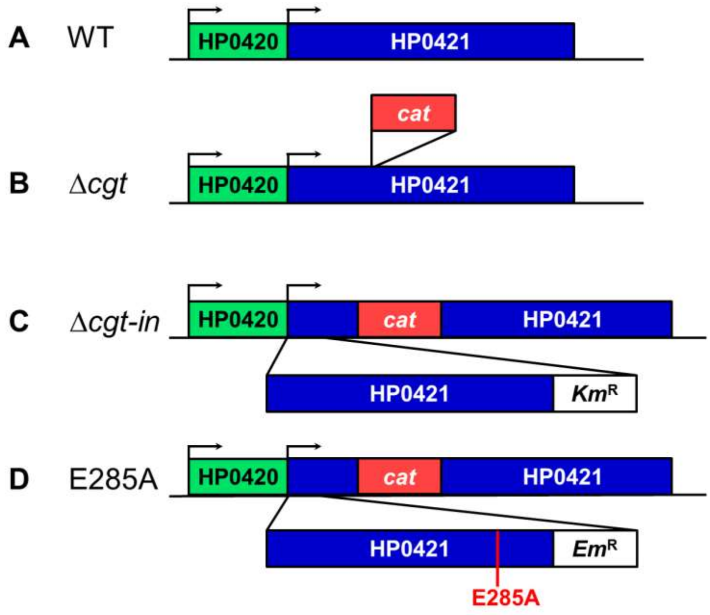


**Figure S1. Construction of isogenic *H. pylori* mutants.** (A) Wild-type *H. pylori* genes with normal *hp0420* and *hp0421* are shown. (B) Chloramphenicol acetyltransferase (*cat*) was inserted into WT *H. pylori hp0421* to generate a *cgt* knockout strain (Δ*cgt*). (C) The full length of *hp0421*, followed by a kanamycin-resistant cassette ($Km^R$) was inserted into Δ*cgt H. pylori hp0421* with to generate a *cgt* knockin strain (Δ*cgt*-in). (D) The full length of *hp0421*, along with a point mutation (E285A) followed by an erythromycin-resistance cassette ($Em^R$) was inserted into Δ*cgt H. pylori hp0421* with to generate a *cgt*-dead mutant strain (*cgt*-dead mutant). E285A: glutamate was mutated and changed to alanine at the amino acid sequence of 285, which is indicated in red.

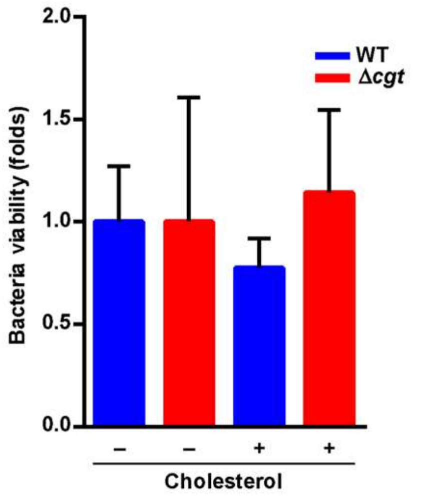


**Figure S2. Exogenous cholesterol coating *H. pylori* does not affect bacterial viability.** WT and Δ*cgt* were incubated with water-soluble cholesterol (5 mg/ml) for 1 h. The cholesterol-treated bacteria were plated by serial dilution onto blood agar plates and incubated for 5 days and the CFUs of viable *H. pylori* were counted.

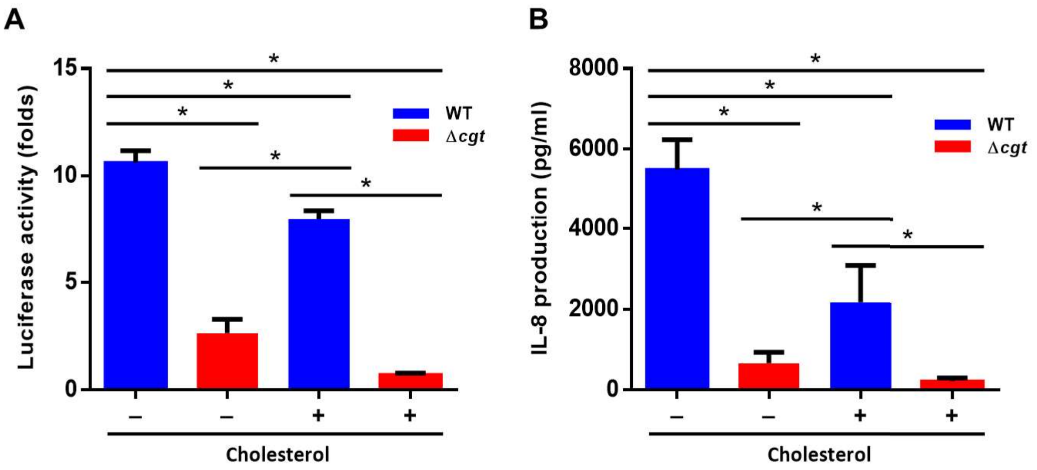


**Figure S3. Excess exogenous cholesterol dampens *H. pylori* adhesion to cells.** *H. pylori* was pretreated with water-soluble cholesterol (5 mg/ml) for 1 h prior to infecting AGS cells at an MOI of 100 for 6 h. (A) The level of NF-κB activation was determined by luciferase assay. (B) IL-8 production was assessed using ELISA. *, $P < 0.05$.
